# Meta-Analysis of Longitudinal Cohort Studies of Suicide Risk Assessment among Psychiatric Patients: Heterogeneity in Results and Lack of Improvement over Time

**DOI:** 10.1371/journal.pone.0156322

**Published:** 2016-06-10

**Authors:** Matthew Large, Muthusamy Kaneson, Nicholas Myles, Hannah Myles, Pramudie Gunaratne, Christopher Ryan

**Affiliations:** 1 School of Psychiatry, University of New South Wales, Randwick, Australia; 2 Prince of Wales Hospital, Randwick, Australia; 3 Faculty of Medicine, University of New South Wales, Randwick, Australia; 4 The Queen Elizabeth Hospital, Woodville South, Australia; 5 Discipline of Psychiatry, School of Medicine, The University of Adelaide, SA, Australia; 6 Country Health SA Mental Health, SA, Australia; 7 Discipline of Psychiatry and Centre for Values Ethics and the Law in Medicine, University of Sydney, Sydney, Australia; 8 Department of Psychiatry, Westmead Hospital, Westmead, NSW, Australia; University of Toronto, CANADA

## Abstract

**Objective:**

It is widely assumed that the clinical care of psychiatric patients can be guided by estimates of suicide risk and by using patient characteristics to define a group of high-risk patients. However, the statistical strength and reliability of suicide risk categorization is unknown. Our objective was to investigate the odds of suicide in high-risk compared to lower-risk categories and the suicide rates in high-risk and lower-risk groups.

**Method:**

We located longitudinal cohort studies where psychiatric patients or people who had made suicide attempts were stratified into high-risk and lower-risk groups for suicide with suicide mortality as the outcome by searching for peer reviewed publications indexed in PubMed or PsychINFO. Electronic searches were supplemented by hand searching of included studies and relevant review articles. Two authors independently extracted data regarding effect size, study population and study design from 53 samples of risk-assessed patients reported in 37 studies.

**Results:**

The pooled odds of suicide among high-risk patients compared to lower-risk patients calculated by random effects meta-analysis was of 4.84 (95% Confidence Interval (CI) 3.79–6.20). Between-study heterogeneity was very high (I^2^ = 93.3). There was no evidence that more recent studies had greater statistical strength than older studies. Over an average follow up period of 63 months the proportion of suicides among the high-risk patients was 5.5% and was 0.9% among lower-risk patients. The meta-analytically derived sensitivity and specificity of a high-risk categorization were 56% and 79% respectively. There was evidence of publication bias in favour of studies that inflated the pooled odds of suicide in high-risk patients.

**Conclusions:**

The strength of suicide risk categorizations based on the presence of multiple risk factors does not greatly exceed the association between individual suicide risk factors and suicide. A statistically strong and reliable method to usefully distinguish patients with a high-risk of suicide remains elusive.

## Introduction

It is widely assumed that patients presenting to psychiatric services should routinely undergo a suicide risk assessment in order to allow the identification of high-risk patients who warrant closer monitoring and who should be afforded more clinical resources [1–4]. However, some authors doubt that clinically meaningful suicide risk categories can be defined by either suicidal thoughts or behaviors [5,6] or a combination of multiple risk factors [7–10]. Complicating this debate is a lack of knowledge about the statistical strength of suicide risk categorization, the extent to which this statistical strength varies between studies or whether there has been genuine progress in this area of research over time.

Numerous longitudinal cohort studies published in the last four decades have defined suicide risk categories or strata by combining various clinical and socio-demographic risk factors. These studies are of two types. The first type, which we will term ‘exploratory’ studies, combine potential risk factors observed at baseline to develop a post-hoc risk model based on eventual suicide at follow-up [7,11,12]. Exploratory studies can consider large numbers of potential risk factors and employ statistical methods such as multiple logistic regression and survival analysis to determine variables that are independently associated with suicide. As a consequence of examining a large number of variables some statistical associations with suicide will arise purely by chance. If these chance associations are incorporated into multivariate high-risk models generated by these studies, the apparent strength of the models can be artificially inflated [8,13,14]. The second type of study, which we will term ‘validation’ studies, determine risk categories through the use of scales that are either previously published or that combine a defined set of variables identified in previous exploratory studies [15–17]. Validation studies typically examine a more limited set of variables than exploratory studies but are less prone to chance findings.

### Aims and hypotheses

We performed a meta-analysis incorporating both exploratory and validation longitudinal cohort studies. Our primary aim was to calculate overall strength of the effect size of suicide risk assessment using a pooled estimate of the odds of suicide in high-risk groups compared to lower-risk groups. We hypothesized that the effect size associated with suicide risk assessment would i) be reliable between studies and resulting in low between study heterogeneity and ii) have improved over time with stronger in results in more recent studies.

Secondary aims were to explore potential moderators of between-study heterogeneity in the primary research according to the methods employed, the type of patients included, the overall strength of reporting, the base rate of suicide, the year of publication, the duration of follow up and the number of independent variables that were examined. Finally we aimed to examine the performance of high-risk models by calculating the proportions of suicides in high-risk groups (positive predicative value) and lower-risk groups, and to calculate the aggregate sensitivity and specificity of risk categorization.

## Methods

We meta-analyzed published longitudinal cohort studies that examined multiple patient factors in order to define a stratum of psychiatric patients at high risk of suicide. ‘Psychiatric patients’ here refers to persons, who received inpatient or outpatient psychiatric treatment, or persons who were assessed after a suicide attempt or an act of deliberate self-harm. Mental health professionals commonly assess both of these patient groups and the suicide rate in both groups is comparatively higher than in the general population [18,19].

We chose to examine longitudinal cohort studies. We did not consider case-control studies because of their potential for bias in data collection of some variables, because of the potential bias associated with retrospective variable selection following suicide outcomes, and because these studies do not allow direct calculation of suicide rates according to risk category. Both exploratory and validation studies were included to provide a complete analysis of suicide risk categorization and allow statistical comparison of study type. When a study reported both exploratory and validation approaches both were included in the meta-analysis.

Our methods conformed to the items in the quality checklist from the Preferred Reporting Items for Systematic Reviews and Meta-Analyses (PRISMA) and Meta-analysis Of Observational Studies in Epidemiology (MOOSE) guidelines [20,21]. PRISMA checklist, see S1 PRISMA Checklist.

### Search strategy

**Fig 1:**
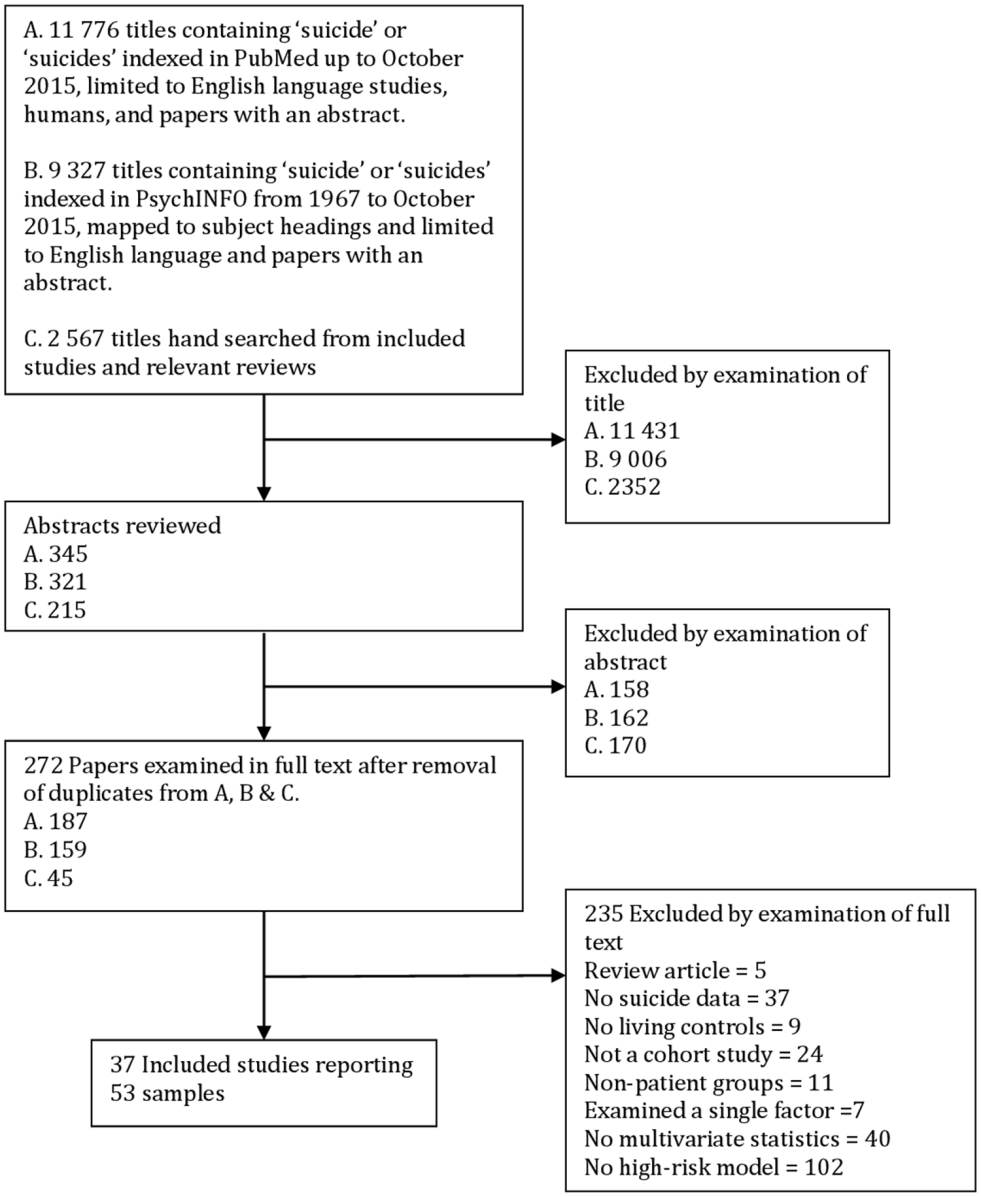
Flow chart of searches for cohort studies reporting multivariate models of later suicide.

Extensive preliminary literature searches using broad subject headings proved insufficiently sensitive to identify several studies known to the authors. Consequently, we conducted less specific searches using the term ‘suicide’ or ‘suicides’ in the title. Two such searches were independently conducted of PubMed and PsycINFO. The searches were conducted in English. Studies were assessed by the inclusion and exclusion criteria below and winnowed first by examination of title, then abstract, then full text,see Fig 1. The reference lists of all the included studies were then hand searched first by examination of relevant titles, abstracts and full text publications without language selection. Finally, we searched the reference list of relevant clinical guidelines,[22,23] books,[24,25] and review articles [6,8,13,26–32].

### Study selection

#### Inclusion criteria

We included studies that: i) reported on longitudinal cohorts of psychiatric patients; ii) measured patient factors at baseline assessment (either in a validation study of a suicide risk scale or in an exploratory study of multiple variables); iii) reported subsequent deaths by suicide as the dependent variable and; iv) used two or more variables (other than age and sex) to define a high suicide risk group.

#### Exclusion criteria

We excluded studies that: were retrospective case controlled studies; reported on suicide attempts as the dependent variable; examined general populations rather than patient groups; did not describe a high-risk group or; described a high-risk group on the basis of a single characteristic or solely demographic characteristics. We also excluded studies that examined potential biological markers because of our focus on clinical practice.

### Data extraction

Two authors (ML and HM) independently extracted the data. The preferred data format was the number of suicides within high-risk and lower-risk groups, and total number within each group. This was imputed from reported sensitivities and specificities in some circumstances. Most studies dichotomized the patients into high and lower-risk groups. Where multiple cut-off points were reported, the data with the highest proportion of suicides in the high-risk group was used in the meta-analysis. Where data reporting suicides and total numbers in risk groups was not available we extracted other effect size data including odds ratios or chi-square statistics. All effect size data were converted to odds ratios.

Moderator variables were collected for each sample comprising:

1. Whether the study was a validation or an exploratory study, because of the possibility of chance findings inflating the statistical power with the latter methodology.
2. Whether the cohort consisted exclusively of people who had made a suicide attempt or subjects recruited from general psychiatric settings.
3. The year the study was published, because suicide risk categorization might have improved over time.
4. The number of potential suicide risk variables initially examined because studies including a larger number of variables are more prone to chance associations.
5. The number of variables in the high-risk model because more detailed risk categories might be more accurate.
6. The mean length of follow up (in months), because studies with longer follow up are less likely to misclassify eventual suicide.
7. The base rate of suicide to allow calculation of sensitivity, specificity and positive predictive value (PPV).
8. Whether coronial or mortality database data were used to define the outcome of suicide, because this methodology has more accurate case ascertainment.
9. Whether subjects were recruited from a geographically defined catchment area, because such studies are less prone to bias.
10. Whether the studies examined suicides of current psychiatric inpatients, because, these studies have a short follow up period restricted to the length of stay in hospital, because inpatient psychiatric care might mitigate suicide risk and because these studies used the number of admissions rather than the number of patients as the denominator.

### Data Synthesis

Random-effects meta-analysis was used to calculate pooled estimates of the odds ratio for suicide among those who were assessed as being at high-risk versus lower-risk using Comprehensive Meta-Analysis (CMA; Version 3, Biostat, Englewood NJ). A random-effects model was chosen *a priori* for all analyses because of the differences in study populations and definitions of high-risk strata. Odds ratios were used as the measure of effect size. Between-study heterogeneity in effect size was examined using the I^2^ and with Q-value statistics. Between-group heterogeneity (sensitivity analysis) was examined without assuming a common within-study variance and the significance of between-group heterogeneity was determined with Q-value statistics.

Random-effects meta-regression (method of moments) was used to examine whether the year of publication, the length of follow up, the base rate of suicide, the number of variables initially considered, and the number of variables in the high-risk model, were associated with between-study heterogeneity. The sensitivity and specificity of the risk categorization and the proportion of suicides in the high-risk and lower-risk groups were also calculated using random-effects meta-analysis.

### Assessment of reporting strength

Six moderator variables derived from relevant items of the Newcastle-Ottawa scale for the assessment of reporting strength of non-randomized cohort studies in meta-analyses were collected to use as items in a strength of reporting scale [33]. These variables included whether: the study was a validation study; the study was drawn from a defined catchment area; the suicides were ascertained using mortality databases or coronial findings; the study did not exclusively report inpatient suicides; the study had a length of follow up that was greater than the median length of follow up; the study reported more suicides than the median number of suicides reported. Any study that featured one of these characteristics was awarded one point, allowing each study to be awarded a maximum of six points.

Validation studies were regarded as higher quality than exploratory studies because of the reduced possibility of variables being included in high-risk models by chance [8]. Studies that reported mortality data or coronial records were regarded as having stronger methodology because of more accurate case ascertainment [34]. Studies with a longer period of follow up were regarded as having stronger methodology due to the decreased likelihood of misclassifying survivors who may eventually suicide. Studies reporting on fewer suicides were regarded as being lower quality because of the increased possibility of chance findings. Studies that did not exclusively examine suicides by psychiatric inpatients were considered to have stronger methodology, because inpatient psychiatric care might mitigate suicide risk and because these studies used the number of admissions rather than the number of patients as the denominator.

### Assessment of publication bias

Publication bias was assessed using an Eggers regression test and with Duval and Tweedie’s trim and fill method [35].

### Sensitivity analyses

Three sensitivity analyses were performed to compare: i) validation studies to exploratory studies; ii) studies that examined patients who presented with deliberate self harm and/or suicide attempts to studies of other psychiatric patients; iii) studies with a higher versus lower total strength of reporting score dichotomized by the median score.

## Results

### Searches

The searches identified 37 relevant studies, see Table 1, reporting 53 samples of patients who were categorized by suicide risk assessment, see S1 File. There was one disagreement about the selection of one included study that was resolved by consensus. There were no disagreements with regard to independently extracted effect size data. Disagreements about 15 (2.2%) of 689 data points in relation to study methods or other moderator variables were resolved by further examination and consensus. In no case was it deemed necessary to contact the authors of the primary research for further clarification of their data.

**Table 1:** Summary of included studies.

Study	Setting and population	Number, suicides and base rate	Follow-up (months)	Variables examined	Study type and items used in high-risk category
1. Buglass & McCulloch (1970)[42]	Patients admitted to Poisoning Treatment Centre of the Royal Infirmary, Edinburgh	541 patients, 17 suicides	36	34 variables with score derived from 3 variables in males and 9 variables in females significantly associated with suicide on univariate analysis	Exploratory. Alcohol use, violent suicide attempt, recent separation (male), previous or current psychiatric treatment, history of self harm, psychopathy, drug addiction, unstable accommodation, poor relationship with children, poor work record and childhood separation (female)
2. Buglass & Horton (1974)[36]	Patients admitted to Poisoning Treatment Centre of the Royal Infirmary, Edinburgh	2603 patients, 21 suicides	12	24 variables with score derived from six variables significantly associated with suicide on univariate analysis	Validation and exploratory. Sociopathy, alcohol abuse, prior inpatients treatment, outpatient treatment, prior suicide attempt, not living with relative
3. Rosen (1976)[43]	Patients admitted for suicide attempt to the Regional Poisoning Treatment Centre of the Edinburgh Royal Infirmary, UK.	876 patients, 34 suicides	60	8 variables with 2 variables selected based on univariate analysis	Exploratory. High-risk defined as having both a medically and psychiatrically serious suicide attempt at admission
4. Pokorny (1983)[7,44]	Inpatients admitted for any psychiatric condition at 9 wards of the Veterans Administration Medical Centre, Houston, US.	4,800 patients, 67 suicides	60	21 variables in initial analysis with second analysis of 4 factors selected on logistic regression	Validation and exploratory. Prior suicide attempt, suicidal ideation, affective disorder, schizophrenia, violence, social withdrawal, urge to do dangerous activities, remorse, fear of loss of control, inpatient treatment, feelings of failure, depressed mood
5. Pallis (1984)[45]	Outpatients referred by three hospitals or community teams following suicide attempts. In Chinchester, East Glamorgan and Southampton, UK.	1,283 patients, 27 suicides	24	SIS short version, SIS long version, SIS-modified	Validation. Predetermined cutoffs
6. Beck (1985)[46]	Patients considered suicidal admitted to Hospital or the University of Pennsylvania General Hospital	165 patients, 11 suicides	72	BHS, BDI, SSI	Validation. Predetermined cut-offs
7. Motto (1985)[47]	Patients admitted for treatment of depressive or suicidal states across 9 psychiatric hospitals in San Francisco, US.	2,753 patients, 136 suicides	24	162 variables with 15 selected based on univariate analysis with optimal cut-off score	Exploratory. Age, skilled employment, wealth, psychiatric history, non-heterosexual, previous psychiatric admissions, failed psychological help, financial strain, social stress, hypersomnia, weight change, persecutory ideas, suicidal impulses, serious suicide attempt, negative counter-transference
8. Clark (1987)[9]	Patients admitted for depressive illness at 5 academic centers in the US	593 patients, 14 suicides	24	15 items identified on Motto’s Risk Estimator for Suicide	Validation. High-risk if greater than the 5^th^ decile of risk
9. Beck & Steer (1989)[48]	Inpatients admitted for recent suicide attempts to Philadelphia General Hospital	413 patients, 20 suicides	74	BDI, BHS, SIS,10 clinical and demographic factors	Validation. Optimal cut-off based on receiver operator curves
10. Beck (1989)[15]	Patients considered suicidal admitted to Hospital of the University of Pennsylvania or Philadelphia General Hospital	141 patients, 10 suicides	84	CHS	Validation. Predetermined cut-offs
11. Allgulander & Fisher (1990)[49]	Patients admitted with intentional psychoactive drug poisoning in Stockholm County, Sweden	8,895 patients, 493 suicides	72	23 clinical and demographic variables using survival analysis	Exploratory. Age, prior attempt, personality disorder, affective disorder, alcohol use, long index admission, prescription drug abuse
12. Motto & Bostrom (1990)[4]	Patients admitted for depressive or suicidal state across 9 psychiatric hospitals in San Francisco, US.	2,999 patients, 38 suicides	2	22 clinical variables with 9 selected on logistic regression with cut-off based on survival analysis	Exploratory. Prior psychiatric hospitalization, consideration of lethal method, suicidal ideas, divorced, financial stress, feeling a burden, negative counter-transference, severe crying or unable to cry, persecutory or referential ideas
13. Goldstein (1991)[10]	Patients admitted with affective disorders to University of Iowa Psychiatric Hospital	1,901 patients, 46 suicides	84	21 clinical variables with final score of 6 significant predictors on logistic regression	Exploratory. Male, suicidal ideation, non-BPAD, unfavorable discharge, unipolar depression with family history of mania, prior suicide attempt
14. Nordentoft (1993)[50]	Patients admitted after suicide attempts by poisoning to Bispebjerg Hospital Denmark.	974 patients, 103 suicides	120	18 variables with models selected by logistic regression and by the presence of more than one risk factor	Exploratory. Male, older age, living alone, more than two or more suicide attempts, depressive psychosis
15. Nordstrom (1995)[51]	Patients currently participating in different psychopharmacological trials of antidepressants from three hospitals in Stockholm consisting of patients with and without suicide attempt at admission.	356 patients, 27 suicides	72	High-risk defined as having suicide attempt at admission with melancholia	Exploratory.
16. Nimeus (1997)[52]	Patients admitted after a suicide attempt to the University Hospital, Lund, Sweden.	212 patients, 13 suicides	52	BHS	Validation. Optimal cutoff based on receiver operator curves
17. Krupinski (1998)[12]	Inpatients admitted to University of Munich Hospital with non-manic affective psychosis.	3,791 patients, 33 suicides	2	272 variables, with 16 variables selected on discriminant function analysis	Exploratory. Suicidal tendency, previous suicide attempt, no early waking, no retarded thinking, no recent inpatient treatment, age, no constipation, more siblings, children, more inpatient treatment, female, current stress, drug or alcohol use, shorter illness
18. Beck (1999)[53]	Outpatients with various psychiatric disorders at Centre for Cognitive Therapy at University of Pennsylvania	3,701 patients, 30 suicides	96	BHS, SSI (worst and current)	Validation. Optimal cut-off based on receiver operator curves
19. Stephens (1999)[54]	Patients admitted with schizophrenia to the Phipps Clinic, Maryland, US.	1,212 patients, 28 suicides	126	35 variables with 7 variables selected based on logistic regression with researcher selected cutoff score	Exploratory. Poor premorbid adjustment, suicidal thoughts, previous suicide attempt, family history of affective illness, current depression, sexual anxiety, psychomotor agitation
20. Tejedor (1999)[55]	Patients admitted to the Psychiatric Department of the Santa Cruz San Pablo Hospital in Barcelona, Spain.	150 patients, 18 suicides	120	32 variables 8 variables selected based on survival analysis	Exploratory. Poor initial and later social function, older age, schizophrenia, previous suicide attempts, suicide attempts during follow up, a past psychiatric history, unemployment
21. Brown (2000)[16]	Outpatients with various psychiatric disorders consecutively evaluated at Centre for Cognitive Therapy at University of Pennsylvania	5,739 patients, 49 suicides	120	BHS, SSI	Validation. Optimal cut-off based on receiver operator curves
22. Krupinski (2000)[38]	Inpatients admitted to University of Munich Hospital with schizophrenia.	5,351 patients, 19 suicides	2	272 variables, with 9 selected on discriminant function analysis	Exploratory. Feeling of numbness, thought insertion, anxiety, depressed mood, anxious depression, suicidal ideation, no delusions, previous suicide attempt, aggression
23. Nimeus (2000)[56]	Patients admitted after a suicide attempt to the Medical Intensive Care Unit of University Hospital, Lund, Sweden.	191 patients, 8 suicides	12	SUAS	Validation. Optimal cutoff based on receiver operator curves
24. Schneider (2001)[57]	Patients admitted to psychiatric hospital in Germany with major depression.	278 patients, 16 suicides	60	4 variables defined on logistic regression	Exploratory. Hypochondriasis, delusions of reference, insomnia, recurrent depression
25. Nimeus (2002)[58]	Patients admitted after a suicide attempt to the Medical Intensive Care Unit of University Hospital, Lund, Sweden.	555 patients, 22 suicides	54	SIS	Validation. Optimal cutoff based on receiver operator curves
26. Skogman (2004)[59]	Patients admitted to the Lund University Hospital, Sweden with suicide attempt.	1,052 patients, 50 suicides	77	SIS score with 3 additional variables for males and 3 additional variables for females based on logistic regression analysis of 11 variables	Exploratory. Suicide repetition, major depression and violent index attempt (male) Age >50 years, major depression and SIS score (females)
27. Suominen (2004)[60]	Patients admitted with attempted suicide to 5 general hospitals in Helsinki, Finland.	224 patients, 17 suicides	144	SIS with 35 additional variables with two selected based on multivariate survival analysis	Exploratory. SIS, physical illness or disability
28. Harriss & Hawton (2005)[61]	Patients admitted with deliberate self-harm in Oxford, UK	2415 patients, 53 suicides	62	SIS combined with 11 variables selected based on significant association with suicide on logistic regression	Exploratory. SIS combined with alcohol misuse (males),SIS combined with age >35 and previous psychiatric treatment (females)
29. Loas (2007)[62]	Patients admitted to Hospital Nord d’Amiens, France with attempted suicide.	106 patients, 7 suicides	78	BDI, rating so of anxiety and two variables selected on survival analysis	Exploratory, Male, anhedonia
30. Neuner (2008)[39]	Inpatients of the Psychiatric University Hospital Regensburg, Germany, multiple diagnoses.	16,755 patients, 41 suicides	1	70 variables with 4 selected based on logistic regression	Exploratory. Treatment resistance, previous suicide attempt, medication side effects, previous psychotherapy
31. Madsen (2012)[37,63]	Inpatients admitted to psychiatric hospitals in Denmark.	126,382 patients, 279 suicides	1	18 clinical variables with 5 selected on logistic regression with high-risk score determined by survival analysis	Exploratory. Affective disorder, previous suicide attempt, recent suicide attempt, outpatient treatment
32. Steeg (2012)[18]	Psychiatric presentations to 5 emergency departments with self-harm in Manchester, Oxford and Derby, UK between 2003–2007, split into exploratory and validation cohorts.	29,571 patients, 92 suicides	6	35 variables with 4 selected based on logistic regression. ReACT	Exploratory and validation. Recent self harm in <1 year, living alone/homeless, cutting as a method of harm, current psychiatric treatment
33. Stefansson (2012)[17]	Patients with recent suicide attempt admitted to Karolinska University Hospital, Stockholm, Sweden.	81 patients, 7 suicides	114	SIS, SIS-modified	Validation. Predetermined cutoffs
34. Rajalin (2013)[64]	Patients followed up after presenting to the Suicide Prevention Clinic at the Karolinska University Hospital, Sweden.	181 patients, 11 suicides	138	8 variables with 2 variables selected based on logistic regression	Exploratory. Family history of suicide, exposure to violence as a child
35. Kessler (2014)[11]	Patients admitted to US Army hospitals for treatment of any psychiatric condition.	53,769 patients, 68 suicides	12	421 variables with 20 variables selected by machine learning survival analysis	Exploratory. Male, older enlistment age, higher military enlistment score, number of registered guns, verbal assault offence, non-violent gun charge, prior suicide attempt, suicidal ideation, outpatient treatment, antidepressant treatment, prior hospitalization, major depression, somatoform disorder, non-PTSD diagnosis, non-affective psychosis, hearing loss
36. Runeson (2015)[65]	Patients presenting after self-harm in Sweden	34,219 patients, 1182 suicides	64	17 diagnostic or suicide attempt related variables	Exploratory. Non-organic psychosis, bipolar disorder, self harm other than poisoning
37. Stefansson (2015) [66]	Patients with recent suicide attempt admitted to Karolinska University Hospital, Stockholm, Sweden.	81 patients, 7 suicides	218	KIVS, KIVS+SIS	Validation. Predetermined cutoffs

Beck Hopelessness Score (BHS), Scale of Suicidal Ideation (SSI), Suicide assessment scale (SUAS), Beck Depression Inventory (BDI), Suicide Intent Scale (SIS), Clinicians Hopelessness Scale (CHS), bipolar affective disorder (BPAD), Karolinska interpersonal violence scale (KIVS) post-traumatic stress disorder (PTSD), ReACT Self Harm Rule (ReACT).

The included papers examined 315,309 people (mean per study 8522, standard deviation (SD) = 22,812, median = 1052) of whom 3114 died by suicide (mean per study 84.2, standard deviation (SD) = 206, median = 27). Eighteen studies recruited patients in psychiatric treatment settings and 19 studies were of patients who had presented after suicide attempts and/or episodes of self-harm. Of the 53 tests of suicide risk categorization 24 were validation studies and 29 were exploratory studies. Three papers reported both validation and exploratory methods [7,18,36]. Four studies were of the suicide of current psychiatric inpatients [12,37–39]. The mean length of study follow up was 64 months (SD = 50, median = 62). Exploratory risk assessments examined an average of 66.8 (SD = 86.9) variables of which 9.2 (SD = 13.6) were included in high-risk models. Validation studies examined 21.9 (SD = 12.7) variables and reported suicide risk scales with an average of 13.9 (SD = 6.4) items. The methods in the studies varied considerably. The Suicidal Intent Scale [40] was most frequently used in validation studies (ten samples). In the 29 exploratory models, a prior history of a suicide attempt was the single most commonly included variable (21 high-risk models), followed by more psychiatric treatment (15 models), a depressed mood or an affective disorder (15 models), and substance use (seven models).

### Meta-analysis

**Fig 2:**
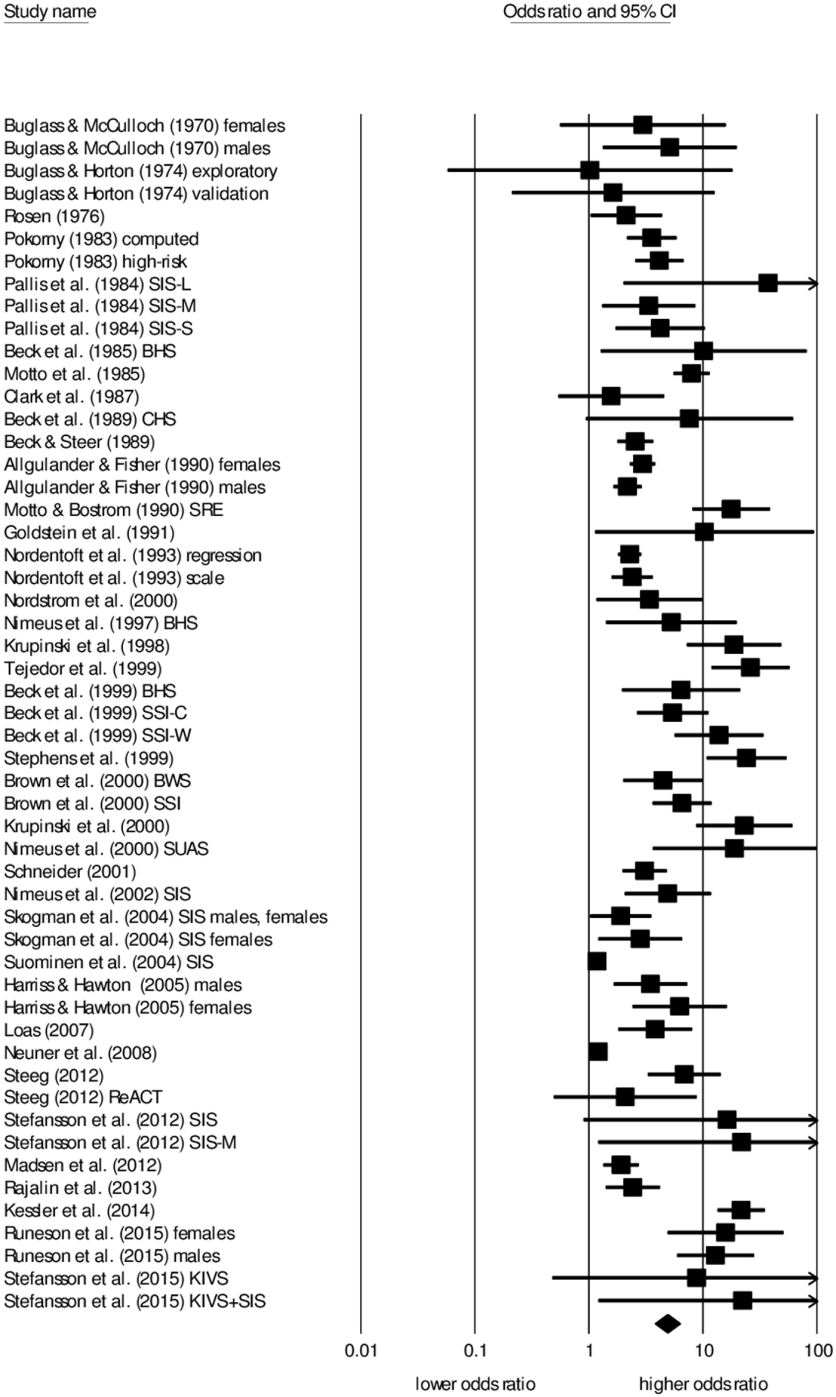
Forrest plot of cohort studies of the odds of suicide in high-risk and lower-risk patients. Studies listed in order of publication. Summary statistic and 95% confidence intervals represented by the diamond. Abbreviations: BHS = Beck Hopelessness Score, SSI = Scale of Suicidal Ideation, SUAS = Suicide assessment scale, BDI = Beck Depression Inventory, SIS = Suicide Intent Scale, SIS-W = Suicide Intent Scale at worst point, SIS-C = Suicide Intent Scale current, SIS-S = Suicide Intent Scale, Short, SIS-L = Suicide Intent Scale, long, SIS-M = Suicide Intent Scale, modified, CHS = Clinicians Hopelessness Scale, KIVS = Karolinska interpersonal violence scale, ReACT = ReACT self harm rule.

The pooled odds of suicide in high risk groups compared to the lower-risk was 4.84 (Table 2 & Fig 2), equivalent to a standardized mean difference 0.87 and indicating a strong effect size) [41]. There was very high between-study heterogeneity (I^2^ = 93.3, Q-value 773, p<0.001). The lowest effect size was an odds ratio of 1.023, the first quartile was an odds ratio of 2.43, the median odds were 4.92, the third quartile was 12.90 and the highest odds ratio was 37.27.

**Table 2:** Meta-analysis of the odds of suicide in high-risk strata compared to other patients.

	Number of samples	Odds ratio	Lower limit	Upper limit	z-value	p-value	Between group heterogeneity
Main analysis (Random effects)	53	4.84	3.79	6.20	12.5	<0.001	
Fixed effects	53	1.60	1.53	1.67	21.3	<0.001	
Exploratory	29	5.13	3.57	7.35	8.88	<0.001	Q-value = 0.09, p-value = 0.76
Validation	24	4.68	2.97	7.40	6.62	<0.001	
Other patient groups	21	6.44	3.70	11.21	6.59	<0.001	Q-value = 2.49, p-value = 0.12
Samples of suicide attempters	32	3.89	2.91	5.20	9.21	<0.001	
Less strong reporting strength	28	4.85	3.54	6.81	9.11	<0.001	Q-value = 0.19, p-value = 0.66
Stronger reporting strength	25	4.41	3.39	5.72	11.1	<0.001	

The 29 samples from studies published before 2000 (odds ratio 4.9, 95% CI 3.7–6.6, I-square = 81) did not from the 24 studies published during or after 2000 (odds ratio 4.6, 95% CI 3.3–5.9, I-square = 93) in either the strength of the effect size or in the extent of between study heterogeneity). Meta regression found that publication date was not significantly associated with effect size of the 53 samples.

There was evidence of publication bias in favor of studies reporting a stronger association between high-risk strata and suicide using Egger’s test (intercept = 3.56, t-value 8.64, two tailed p = 0.001). Duval and Tweedie’s trim and fill method identified 15 hypothetically missing studies with a weaker association between high-risk status and suicide which, if included, would have returned a lower adjusted odds ratio of 3.18 (95% CI 2.55 to 3.99).

The pooled sensitivity of a high-risk categorization was 56% (N = 39 studies, 95% CI 48–64%, I^2^ = 87.5) indicating that just over half of the suicides occurred in the high-risk groups. The pooled specificity of a lower-risk categorization was 79% (95% CI 70–86%, I-Square = 99.9) indicating that four in five of the survivors were in the low risk group. The pooled estimate for the crude suicide rate among high-risk patients (or positive predictive value) was 5.5% (n = 39, 95% CI 3.5–8.5%, I^2^ = 97.4). The pooled rate of suicide in the lower-risk patients was 0.9% (N = 39 samples 95% CI 0.5–1.7%, I^2^ = 98.8).

#### Sensitivity analysis and Meta regression

Validation studies and exploratory studies reported similar pooled effect sizes, both with very high between-study heterogeneity (exploratory, I^2^ = 95.5, Q-value 618, p<0.001; validations, I^2^ = 85.1, Q-value 154, p<0.001). Studies of general psychiatric patients and studies of patients who were recruited after a suicide attempt or an episode of deliberate self-harm had a similar effect size. Studies with a total strength of reporting score of four or more had a similar effect size to studies with a lower strength of reporting score, see Table 2.

The between-study heterogeneity in odds ratios was not explained by the year in which the study was published, the base rate of suicide, the length of follow up, or the number of variables in the high-risk model, see Table 3. A larger number of variables examined at baseline was associated with a stronger effect size but accounted for little of the observed between-study heterogeneity (unexplained variance, I^2^ = 93%).

**Table 3:** Meta-regression examining factors associated with between study heterogeneity in the odds of suicide in high-risk strata.

	Coefficient	Standard error	Lower limit	Upper limit	Z-value	p-value
Year of publication	0.014	0.011	-0.007	0.035	1.27	0.20
Base rate of suicide	-2.61	2.38	-7.28	2.06	-1.09	0.27
Length of follow-up	<0.001	0.003	-0.007	0.007	0.01	0.99
Number of variables initially examined	0.005	0.002	0.002	0.008	2.91	0.004
Number of variables in high-risk model	0.007	0.012	-0.016	0.03	.63	0.53

## Discussion

The pooled estimate from a large and representative body of research conducted over 40 years suggests a statistically strong association between high-risk strata and completed suicide. However the meta-analysis of the sensitivity of suicide risk categorization found that about half of all suicides are likely to occur in lower-risk groups and the meta-analysis of PPV suggests that 95% of high-risk patients will not suicide. Importantly, the pooled odds ratio (and the estimates of the sensitivity and PPV) and any assessment of the overall strength of risk assessment should be interpreted very cautiously in the context of several limitations documented below.

With respect to our first hypothesis, the statistical estimates of between study heterogeneity and the distribution of the outlying, quartile and median effect sizes values suggests that the statistical strength of suicide risk assessment cannot be considered to be consistent between studies, potentially limiting the generalizability of the pooled estimate.

With respect to our second hypothesis we found no evidence that the statistical strength of suicide risk assessment has improved over time.

### Limitations to the generalizability of the pooled estimate

The most important limitation to our pooled estimate is the very high between-study heterogeneity of the effect size. This between-study heterogeneity was not well explained by our predetermined moderator variables or measures of reporting strength. This suggests that our results should not be considered to be generalizable. Moreover, the pooled estimate was potentially influenced by evidence of publication bias towards selective reporting of studies with a stronger effect size. While we cannot know for certain whether this bias is present, or to what extent it might occur, it may be that the results reported here are better than can be readily achieved.

A further limitation, potentially inflating the pooled estimate of the effect size, is that studies that initially examined a larger number of variables tended to have a stronger effect size than studies that examined fewer initial variables. This may be because a more detailed assessment of patient factors might have resulted in a model better able to categorize patient’s suicide risk, or because of the inclusion of more variables with chance associations. However, neither of these explanations seems likely because there was no evidence that studies that used more variables to define the high-risk model had a stronger effect size. In fact, the pooled effect size in this study of risk categories based on multiple variables is similar to the meta-analytically derived effect size of individual factors of self-harm, depressed mood and hopelessness among patient groups [13,29].

### Limitations to the meta-analysis

A number of limitations inherent to both this meta-analysis and the primary literature also warrant discussion. A weakness of this meta-analysis is the inclusion of studies conducted over more than 40 years, during which the studies differed greatly in their sample sizes, methods and in their reporting strength.

Our focus on high-risk patients can also be considered a limitation. The included literature does not allow determination of whether a clinically meaningful low-risk group can be defined based on protective factors. It might be that the high prevalence of non-suicide could allow the identification of a group of patients with low suicide rates that is similar to the general community. Moreover, because our focus was on patients, we did not examine the potential strength of risk categorization for suicide among the general community.

A weakness of the primary literature is that all included studies were naturalistic. As a result, this meta-analysis was unable to consider the impact of any interventions that might have been provided to people who were perceived as being at high-risk of suicide. Successful interventions provided to high-risk patients in the primary studies may have the effect of reducing the odds of suicide in that group. The extent of this effect cannot be estimated without studies that directly investigate the effectiveness of providing increased resource allocation or enhanced clinical surveillance to high-risk patients.

Finally, the meta-analysis does not address the statistical power of imminent suicide risk assessment nor did it examine clinical risk assessment. We found no studies of suicide outcomes over periods of less than a month and no study examined the type of heuristic assessment of suicide risk that is common in clinical practice or the relative performance of this to codified risk assessment tools.

Disappointingly, there was no evidence that heterogeneity in effect size could be explained by the year of study publication. This suggests that generally there might have been little progress over time in the ability of published models to identify high-risk groups of patients. However, the lack of evidence for the development of more accurate risk assessment models over time does not mean that such developments are impossible. More sophisticated or effective methods of suicide risk categorization might be developed in the future. For example, one recent study examining post discharge suicide in the US military was able to define a high-risk group with an odds ratio of 22 when compared to lower-risk patients [11]. This study drew on an extensive data set using sophisticated methods of modeling derived from artificial intelligence research, methods that might be able to more strongly and more reliably define high-risk groups in the future.

## Conclusion

Despite decades of research, the psychometric properties of optimal suicide risk categorization remains uncertain. The extent of this uncertainty is profound and our results are not reassuring. It remains to be seen if methods can be developed to consistently and clearly distinguish high-risk from lower-risk patients. However, it should not be forgotten that the ultimate utility of risk categorization depends on its potential for application. Even a strong statistical discrimination between high and lower-risk groups lacks meaning if there are no rational interventions that should be provided to high risk patients (the vast majority of whom will not suicide) yet should not be given to low risk patients, among whom about half of all suicides might occur. Moreover, ultimately the value of suicide risk categorization must be judged by whether it can actually contribute to a reduction in patient suicide mortality.

## Supporting Information

S1 File Data used in the meta-analysis. (XLSX)

S1 PRISMA Checklist (DOC)
